# Delivery of long-term-injectable agents for TB by lay carers: pragmatic randomised trial

**DOI:** 10.1136/thoraxjnl-2018-212675

**Published:** 2019-11-01

**Authors:** Danielle B Cohen, Kuzani Mbendera, Hendramoorthy Maheswaran, Mavuto Mukaka, Helen Mangochi, Linna Phiri, Jason Madan, Geraint Davies, Elizabeth Corbett, Bertel Squire

**Affiliations:** 1 Infection, Immunity & Cardiovascular Disease, University of Sheffield, Sheffield, UK; 2 Clinical Department, Malawi Liverpool Wellcome Clinical Research Programme, Blantyre, Malawi; 3 Department of Clinical Sciences, Liverpool School of Tropical Medicine, Liverpool, UK; 4 Malawi National TB Programme, Lilongwe, Malawi; 5 Institute of Psychology, Health and Society, University of Liverpool, Liverpool, UK; 6 Division of Health Sciences, University of Warwick, Warwick, UK; 7 Mahidol Oxford Tropical Medicine Research Unit, Mahidol University, Bankok, Thailand; 8 Centre for Tropical Medicine and Global Health, University of Oxford, Oxford, United Kingdom; 9 Institute of Infection and Global Health, University of Liverpool, Liverpool, United Kingdom; 10 Department of Clinical Research, LSHTM, London, UK

**Keywords:** streptomycin, OPAT, community-based care, catastrophic household costs, drug-resistant TB, recurrent TB, retreatment TB

## Abstract

**Background:**

People with recurrent or drug-resistant TB require long courses of intramuscular injections. We evaluate a novel system in which patient-nominated lay carers were trained to deliver intramuscular injections to patients in their own homes.

**Methods:**

A pragmatic, individually randomised non-inferiority trial was conducted at two hospitals in Malawi. Adults starting TB retreatment were recruited. Patients randomised to the intervention received home-based care from patient-nominated lay people trained to deliver intramuscular streptomycin. Patients receiving standard care were admitted to hospital for 2 months of streptomycin. The primary outcome was successful treatment (alive and on treatment) at the end of the intervention.

**Results:**

Of 456 patients screened, 204 participants were randomised. The trial was terminated early due to futility. At the end of the intervention, 97/101 (96.0%) in the hospital arm were still alive and on treatment compared with 96/103 (93.2%) in the home-based arm (risk difference −0.03 (95% CI −0.09 to 0.03); p value 0.538). There were no differences in the proportion completing 8 months of anti-TB treatment; or the proportion experiencing 2-month sputum culture conversion. The mean cost of hospital-based management was US$1546.3 per person, compared to US$729.2 for home-based management. Home-based care reduced risk of catastrophic household costs by 84%.

**Conclusions:**

Although this trial failed to meet target recruitment, the available data demonstrate that training patient-nominated lay people has potential to provide a feasible solution to the operational challenges associated with delivering long-term-injectable drugs to people with recurrent or drug-resistant TB in resource-limited settings, and substantially reduce costs. Further data under operational conditions are required.

**Trial registration number:**

ISRCTN05815615.

**Key messages**

**What is the key question?**

- Can patient-nominated lay carers provide intramuscular injections at home to patients requiring long-term-injectable agents for the treatment of TB?

**What is the bottom line?**

- This model of delivering care to patients receiving daily intramuscular drugs for TB could be feasible and cost saving for both users and providers.

**Why read on?**

- Training lay carers to give intramuscular injections to patients in their own homes may present a novel opportunity to improve the delivery of care to patients with recurrent or drug-resistant TB.

## Background

Each year, approximately 700 000 people are treated for recurrent TB and a further 480 000 for multidrug-resistant TB (MDR-TB).^1^ Treatment regimens for both of these groups currently involve long courses of daily injectable agents. In the case of TB retreatment, patients in countries with low prevalence of MDR-TB and no access to drug susceptibility testing have been prescribed WHO ‘Category II retreatment regimen’.^2^ This is an 8-month course of oral antituberculous agents with the addition of intramuscular streptomycin for the first 60 days. Most patients with MDR-TB still receive at least 8 months of injectables^3^ and even newer shortened regimens for MDR-TB include 4 months of injectable agents.^4^

Traditionally, patients have been admitted to hospital to receive injections, but long admissions are expensive for both users and providers^5–7^ and associated with acquisition of nosocomial infections.^8 9^ More recently, community-based approaches to delivering parenteral drugs have been encouraged. Models of delivery have involved either patients travelling daily from the community to a health facility, or a professional health worker visiting patients in their homes. Both models pose significant operational challenges,^10^ and a potential solution is for injections to be given to patients at home by a person living with them or close by.

Over recent years in industrialised countries, programmes of outpatient parenteral antibiotic therapy have been successfully instituted.^11–13^ Evidence suggests that these services are safe, well received by users and highly cost effective.^14^ Patients with diabetes in non-industrialised countries are prescribed insulin and successfully administer subcutaneous injections at home^15^; however, no model of carer-administered intramuscular treatment has yet been developed in a resource-limited setting.

In Malawi, ‘guardians’ have a well-established essential role in caring for people admitted to hospital. They are usually family members or friends who accompany patients, and perform a variety of tasks including basic care, assisting with medications and advocacy.^16^ The aim of this trial was to evaluate a novel method of delivering long-term injectables for TB in which patient-nominated lay people (guardians) were trained to administer daily intramuscular injections to patients in their own homes.

## Methods

### Trial design and study participants

We conducted a pragmatic, individually randomised trial of hospital versus home-based care during the intensive phase of TB retreatment. Participants randomised to the intervention received home-based care from guardians trained to deliver intramuscular streptomycin. Participants randomised to receive standard care were admitted to hospital for 60 days, as was practice in Malawi.

The trial was conducted at two large hospitals in Malawi—Queen Elizabeth Central Hospital (QECH) in Blantyre and Bwaila Hospital in Lilongwe. Consecutive patients registering for TB retreatment were recruited. Individuals were eligible if they were ≥16 years old, able to provide informed consent and able to identify a suitable guardian. People were excluded if they were identified as having MDR-TB or rifampicin-resistant TB; pregnant; or not planning to stay in the area.

### Study procedures

Standard practice in Malawi at the time of the trial was to admit all patients requiring streptomycin for the duration of the course. Patients admitted to hospital to start retreatment were identified each weekday by reviewing the TB and ward registers. Given the pragmatic nature of this trial, the diagnosis of TB was based on clinician assessment in usual operational conditions. Patients were asked to nominate a guardian for training. Consent was obtained separately from both patients and guardians.

A study nurse trained guardians in the technique of intramuscular injection, including injection procedure, sterile technique and disposal of sharps. Once the guardian was able to perform injections safely, they underwent a structured competency assessment (see online supplementary file 1). Participants remained on the ward until randomisation.

10.1136/thoraxjnl-2018-212675.supp1 Supplementary data

#### Randomisation

Randomisation was performed once the guardian had passed the competency assessment and the patient was fit for discharge. Participants were randomised to receive standard of care (hospital admission) or the intervention (home-based care) during the intensive phase of TB retreatment. Randomisation was carried out by a data team not otherwise involved in the conduct of the trial. Block randomisation in a ratio of 1:1 using variable block sizes of 4 or 6 was performed using a computer random number generator to produce an equal allocation ratio. Sequentially numbered opaque sealed envelopes were prepared by an independent person.

#### Follow-up of study participants

Participants in both arms were reviewed 1, 3, 5 and 7 weeks post randomisation. Reviews were conducted by a fieldworker with no formal clinical training. At each visit, an assessment for adverse events was performed, including assessments of hearing^17^; urine output; sciatic nerve injury; inspection of the injection site and documentation of new prescribed medications. For participants in the community, all used equipment was collected, and new equipment was delivered. At the first visit, guardians were required to pass another competency test to ensure competency was maintained in the home environment. Adherence was assessed at each visit by self-report and ‘vial count’ of streptomycin, equivalent to ‘pill count’ employed to assess adherence to antiretroviral therapy (ART) at HIV clinics in the region. Participants were provided with a form and asked to document each day that streptomycin had been given (see online supplementary file 2), in addition to the ‘TB card’ provided routinely for monitoring oral treatments. There was no formal assessment of adherence to HIV medications.

10.1136/thoraxjnl-2018-212675.supp2 Supplementary data

#### Adverse events monitoring

Given the nature of the intervention, standard definitions were adapted to encompass all possible consequences of home-based care (see online supplementary file 3). All patients presenting with clinical adverse events were reviewed by a study clinician (see online supplementary file 4).

10.1136/thoraxjnl-2018-212675.supp3 Supplementary data

10.1136/thoraxjnl-2018-212675.supp4 Supplementary data

### Outcomes

The primary outcome was successful treatment at the end of the 2-month intervention period. Successful treatment was defined as all those ‘still alive and on treatment having completed 2 months streptomycin injections’. Unsuccessful treatment included all patients died, lost to follow-up or outcome unknown.^18^

Predefined secondary endpoints included 2-month sputum culture conversion; programmatic TB outcome after 8 months of treatment; Karnofsky score at 2 months and mental health status at 2 months using a standard Self-Reporting Questionnaire (SRQ) validated in the local language.^19^

### Statistical analysis

The study was powered to detect non-inferiority in the intervention arm. It was assumed that 87% of patients receiving standard of care and 82% of patients in the community would be alive and on treatment at the end of the 60-day intervention period, based on a review of records we performed of QECH data in 2012. The sample size was calculated based on a non-inferiority margin of 6%. Using a one-sided alpha at a level of 0.05, in order to achieve a power of 80%, it was calculated that a sample size of 268 would be required. A single planned interim analysis was conducted after 130 had been recruited.

Primary analysis was based on intention to treat. Secondary per-protocol analysis was planned as a sensitivity analysis, and for this purpose protocol deviation was defined as any participant randomised to receive home-based management who was not discharged by the time they completed 60 days of streptomycin.

Efficacy outcomes are presented as proportions and compared using the Fishers exact test. The risk difference (RD) for unsuccessful treatment outcome was estimated with 95% CIs. Risk ratios (RRs) with 95% CIs are presented for key secondary outcomes. There were no planned subgroup analyses. Analysis was performed using STATA V.12.1.

A within-trial cost-consequence analysis^20^ was undertaken from the societal perspective and included provider and user costs (see online supplementary file 5). Catastrohic cost was defined as healthcare costs equivalent to 10% of annual household income. Written informed consent was obtained from all study participants if literate, or with the signature of an independent witness if illiterate.

10.1136/thoraxjnl-2018-212675.supp5 Supplementary data

## Results

### Study participants

**Figure 1:**
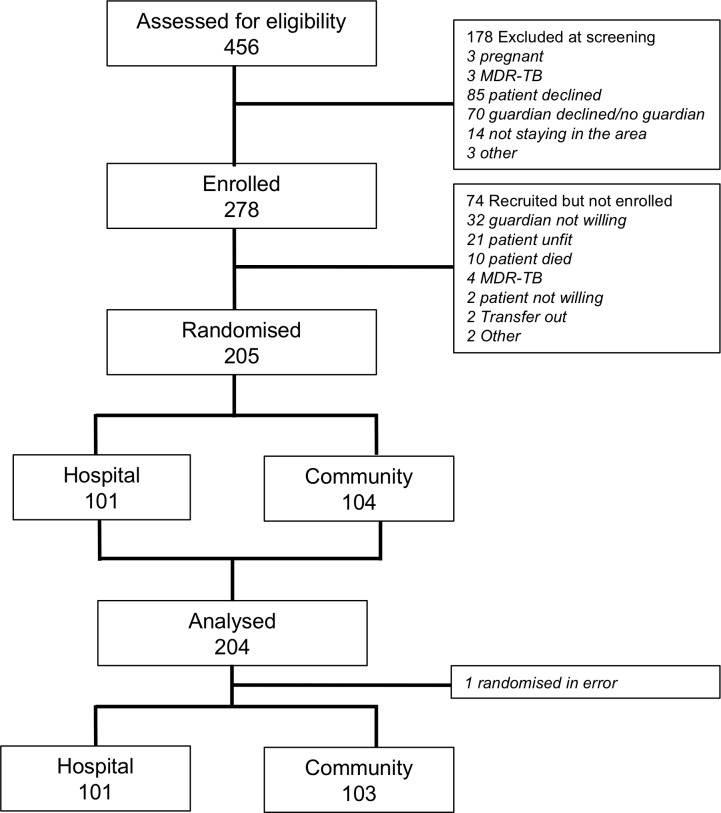
Trial recruitment. MDR-TB, multidrug-resistant TB.

Between June 2013 and February 2015, 456 patients starting TB retreatment regimen were screened. Of those screened, 278 (60%) had guardians who began training to administer streptomycin. After further withdrawals during the period of training, a total of 204 participants were randomised (figure 1). One patient was excluded from the analysis as they were randomised in error prior to being declared fit for discharge by a clinician. One hundred and forty-four were enrolled in Blantyre and the remaining 60 in Lilongwe.

A meeting of the Trial Steering Committee was held after 200 patients had been recruited, at which it was decided to stop recruitment of the trial early due to futility. The event rate in the trial was lower than the 0.13 which had been projected: 0.0645 in the intervention arm and 0.0426 in the control arm, so it was projected that in order to establish non-inferiority, at least 600 participants would be required, and that this was not feasible given the resources available.

Characteristics of the study participants are summarised in table 1. The median age of patients was 36.5 years (IQR 30.0–44.0 years), and 69.6% were men. The majority (89.2%) had pulmonary TB, classified most commonly as relapse (48.5%) or ‘other’ (41.7%). The HIV prevalence was 80.4% and 131/164 (80.9%) were on ART. The median length of time from start of TB treatment to discharge in the home-based group was 12 days (IQR 8–17 days).

**Table 1:** Baseline characteristics of study participants

	Total study population n=204 (%)	Hospital-based management n=101 (%)	Home-based management n=103 (%)
Patient characteristics			
Patient age (median, IQR)	36.5 (30.0–44.0)	37.0 (30.0–44.0)	36.0 (30.0–43.0)
Patient sex (% male)	142 (69.6)	77 (74.8)	65 (64.4)
TB class			
Pulmonary	182 (89.2)	84 (83.2)	98 (95.2)
Extrapulmonary	22 (10.78)	17 (16.8)	5 (4.8)
TB category*			
Relapse	99 (48.5)	44 (43.6)	55 (53.4)
TALTFU	5 (2.5)	2 (2.0)	3 (2.9)
Fail	15 (7.4)	5 (5.0)	10 (9.7)
Other	85 (41.7)	50 (49.5)	35 (34.0)
Sputum culture positive	80 (39.2)	38 (37.6)	42 (40.8)
HIV positive	164 (80.4)	87 (86.1)	77 (74.8)
Established on ART if HIV positive	131 (80.9)	70 (81.4)	61 (80.26)
CD4 count (cells/mm^3^; median, IQR)	177 (82–423)	161 (68–339)	225 (117–560)
No of previous TB episodes			
1	174 (85.3)	88 (87.1)	86 (83.5)
2	28 (13.7)	12 (11.9)	16 (15.5)
>2	2 (1.0)	1 (1.0)	1 (1.0)
Baseline Karnofsky Score (median, IQR)	90 (80–100)	90 (80–90)	90 (90–100)
History of alcohol excess†	47 (23.7)	21 (21.1)	26 (26.3)
Guardian characteristics			
Guardian age (median, IQR)	32 (26–40)	33 (28–39)	30 (25–40)
Guardian sex (% male)	62 (30.4)	28 (27.7)	34 (33.0)
Guardian level of education			
None	4 (2.0)	4 (4.0)	0 (0.0)
Standard 1–4	33 (16.2)	20 (19.8)	13 (12.6)
Standard 5–8	52 (25.5)	26 (25.7)	26 (25.2)
Form 1–2	42 (20.6)	18 (17.8)	24 (23.3)
Form 3–4	45 (22.1)	20 (19.8)	25 (24.3)
University	28 (13.7)	13 (12.9)	15 (14.6)
Guardian relationship to patient			
Spouse	74 (36.3)	37 (36.6)	37 (35.9)
Sibling	56 (27.5)	28 (27.7)	28 (27.2)
Parent	16 (7.8)	9 (2.9)	7 (6.8)
Child	19 (9.3)	7 (6.9)	12 (11.7)
Aunt/uncle	14 (6.9)	7 (6.9)	7 (6.8)
Niece/nephew	9 (4.4)	3 (3.0)	6 (5.8)
Friend/neighbour	16 (7.8)	10 (9.9)	6 (5.8)

*Standard WHO definitions of TB category: relapse=a patient previously treated for TB, declared cured or treatment completed, who is diagnosed again with smear or culture-positive TB; treatment after failure=a patient who is started on retreatment regimen after having failed previous treatment; treatment after loss to follow-up (TALTFU)=a patient who returns to treatment, positive bacteriologically, following interruption of treatment for 2 or more consecutive months; other previously treated=all previously treated cases that do not fit any of the above definitions http://www.who.int/tb/err/rr_final_forms_en.pdf.

†As defined by the Alcohol Use Disorders Identification Test.^36^

### Successful completion of the intensive phase of TB treatment

At the end of the intensive phase of treatment, 97 (96.0%) of patients in the hospital arm and 96 (93.2%) of patients in the home-based arm were still alive and on treatment (RD −0.03 (95% CI −0.09 to 0.03); p value 0.538). Only an intention-to-treat analysis was performed, as all randomised patients received the form of treatment to which they are assigned. There were seven deaths in the home-based arm, and two in the hospital arm (RR 3.43; 95% CI 0.73 to 16.13) (table 2). Deaths were reviewed at a meeting of the Data and Safety Monitoring Board, and it was concluded that none were either directly or indirectly related to the intervention. Of the seven deaths which occurred in the home-based arm, six occurred after the patient had been readmitted to hospital (table 3). Two patients were lost to follow-up from hospital-based care, but none were lost to follow-up from home-based care.

**Table 2:** TB retreatment outcomes at 2 months

	Hospital-based management n=101 (%)	Home-based management n=103 (%)	P value	RR (95% CI)
Alive and on TB treatment	97 (96.0)	96 (93.2)	0.538	0.97 (0.91 to 1.03)
Death	2 (2.0)	7 (6.8)	0.170	3.43 (0.73 to 16.13)
Loss to follow-up	2 (2.0)	0 (0.0)	0.244	Not applicable

RR, risk ratio.

**Table 3:** Details of deaths during the intensive phase of TB retreatment

Details of event	Location
**Home-based treatment arm**	
Stevens Johnsons syndrome 2 weeks after starting TB treatment, 1 week after starting ART	Hospital
End stage heart failure with dilated cardiomyopathy; on treatment for presumed TB pericarditis	Hospital
Acute febrile illness with jaundice; treated for presumed bacterial sepsis	Hospital
Disseminated Kaposi Sarcoma, pancytopenia, ascites, splenomegaly	Hospital
Headache, dysphasia, hemiparesis; treated for presumed cerebral toxoplasmosis	Hospital
Cryptococcal meningitis	Hospital
Sudden death	Community
**Hospital-based treatment arm**	
Died while receiving bowel prep for colonoscopy; presumed electrolyte imbalance	Hospital
Jaundice; presumed drug-induced liver injury	Hospital

### Secondary outcomes

#### End of TB treatment outcomes

Before the close of the study, 162 patients had registered TB treatment outcomes through the National TB Programme. There was no significant difference in the proportion of patients who successfully completed treatment in the home-based arm (80.7%) compared with the hospital arm (77.2%) of the trial (table 4).

**Table 4:** TB retreatment outcomes at 8 months

	Hospital-based management n=79 (%)	Home-based management n=83 (%)	P value	RR (95% CI)
Successful treatment	61 (77.2)	67 (80.7)	0.700	1.05 (0.89 to 1.23)
Cure	26 (32.9)	41 (49.4)	0.039	1.50 (1.02 to 2.20)
Complete	35 (44.3)	26 (31.3)	0.106	0.71 (0.47 to 1.06)
Fail	1 (1.2)	1 (1.2)	1.000	0.95 (0.06 to 14.96)
Death	11 (13.9)	13 (15.7)	0.827	1.12 (0.54 to 2.36)
Loss to follow-up	4 (5.1)	2 (2.4)	0.434	0.48 (0.09 to 2.53)
Transfer out	2 (2.5)	0 (0.0)	0.236	Not applicable

RR, risk ratio.

#### Two-month sputum culture conversion

Of 80 patients who had sputum culture positive for *Mycobacterium tuberculosis* at the start of treatment, sputum culture was performed in 43 who were able to provide a sample after 2 months of treatment. In the group receiving home-based care, 21/23 (91.3%) went from sputum culture positive to sputum culture negative at 2 months, compared with 15/20 (75.0%) who culture converted in the hospital-based care group (RR 1.22 (95% CI 0.91 to 1.61); p value 0.222).

#### Karnofsky score at the end of the intensive phase of treatment

The median Karnofsky score at the end of the intervention period was 100% in both groups. Of patients still alive at the end of the intensive phase of treatment, 84.4% in the home-based group and 79.2% in the hospital group had a Karnofsky score of 100%.

#### Mental health status at the end of the intensive phase of treatment

All but one of the 21 patients identified as having common mental health disorder at baseline no longer screened positive on the SRQ after 2 months of treatment. The patient who continued to screen positive had received hospital-based care.

### Adverse events

There were 56 adverse events in 36 patients receiving hospital-based care and 34 adverse events in 20 patients receiving home-based care (table 5). One patient reported a missed dose of streptomycin and two patients missed a single dose by ‘vial count’, but there was no episode which met the predefined criteria for an adherence event. There were no needle stick injuries in either arm. There were eight serious adverse events in the hospital arm and five in the home-based arm. Both adverse events resulting in disability were a consequence of ototoxicity.

**Table 5:** Adverse events during the intensive phase of TB retreatment

	Hospital-based management n=101	Home-based management n=103
Total adverse events	56	34
Serious adverse events	8	5
Life-threatening illness	6	0
Hospitalisation	0	5
Significant disability	2	0
Needle stick injury	0	0

### Economic evaluation

Data about user costs were available for 188 trial participants, and data about provider costs were collected for a subgroup of 65 participants (32 receiving home-based care and 33 receiving hospital-based care).

The total cost of hospital-based management was US$1546.3 per person compared with US$729.2 per person who received home-based management, giving a cost difference of US$ −817.1 (table 6). The total mean cost to users in the hospital arm was US$271.6 (234.4–308.7), compared with US$101.8 (85.1–118.4) for those who receiving home-based care (mean difference 169.8; 95% CI 128.9 to 210.7) (table 7). Total provider costs were US$498.0 (425.6–570.4) per patient who received home-based care, compared with US$1100.3 (1040.8–1159.8) per patient who received hospital-based care (see online supplementary file 6).

10.1136/thoraxjnl-2018-212675.supp6 Supplementary data

**Table 6:** Costs of home-based management in the intensive phase of TB retreatment

	Hospital-based management n=33	Home-based management n=32
**Cost indicators** Total cost 2014 US$	47 936.6	21 875.6
**Outcome indicators** Number of patients completing intensive phase retreatment	31	30
**Cost-difference indicators** Cost per patient completing intensive phase retreatment 2014 US$ *	1546.3 (833.8–2764.1)	729.2 (341.5–1589.1)
**Incremental cost difference** Cost per patient completing intensive phase retreatment 2014 US$	−817.1	

**Table 7:** Total user costs of hospital-based and home-based management during the intensive phase of TB retreatment

	Mean cost US dollars (95% CI)		
	Hospital-based management	Home-based management	Mean difference*
Patient costs			
During admission			
Direct medical†	0.7 (0.2 to 1.2)	0.4 (0.0 to 0.7)	0.3 (−0.2 to 1.0)
Direct non-medical			
Food	69.8 (58.4 to 81.2)	26.7 (21.5 to 31.8)	43.1 (30.5 to 55.7)
Transport	3.6 (1.6 to 5.7)	1.6 (0.5 to 2.6)	2.1 (−0.1 to 4.3)
Linen	0.7 (0.1 to 1.3)	1.1 (−0.1 to 2.3)	−0.5 (−1.8 to 0.9)
Other out of pocket	11.9 (7.1 to 16.7)	4.7 (3.2 to 6.2)	7.2 (2.1 to 12.4)
Indirect‡	133.5 (111.6 to 155.4)	37.0 (28.7 to 45.3)	96.5 (73.2 to 119.7)
Health facility post discharge			
Direct medical and non-medical	–	1.1 (0.3 to 2.0)	−1.1 (−2.0 to -0.3)
Indirect	–	0.3 (0.1 to 0.5)	−0.8 (−1.8 to 0.1)
Total patient costs	220.2 (189.8 to 250.8)	72.9 (60.5 to 85.3)	147.4 (114.1 to 180.6)
Guardian costs			
During admission			
Direct non-medical			
Food	17.7 (11.1 to 24.2)	13.6 (9.2 to 17.9)	4.1 (−3.5 to 11.7)
Transport	10.0 (5.6 to 14.3)	6.4 (4.1 to 8.8)	3.5 (−1.2 to 8.2)
Linen	0.1 (−0.1 to 0.2)	0.1 (0.0 to 0.3)	0.0 (−0.3 to 0.2)
Other out of pocket	2.9 (1.1 to 4.7)	1.6 (0.7 to 2.5)	1.3 (−0.7 to 3.4)
Indirect	20.6 (8.4 to 32.9)	5.2 (2.8 to 7.7)	15.4 (3.1 to 27.7)
Health facility post discharge			
Direct non-medical	–	1.0 (−0.6 to 2.7)	−1.0 (−2.7 to 0.6)
Indirect	–	0.8 (−0.1 to 1.8)	−0.8 (−1.8 to 0.1)
Total guardian costs	51.3 (31.8 to 70.8)	28.6 (21.5 to 36.2)	22.4 (2.2 to 42.7)
Total user costs	271.6 (234.4 to 308.7)	101.8 (85.1 to 118.4)	169.8 (128.9 to 210.7)

*Bootstrapped estimates of mean differences and 95% CI.

†Drugs and investigations purchased privately if unavailable in the public facility.

‡Lost income.

The risk of catastrophic household costs was 34.1% for participants in the home-based arm and 85.9% for those managed in hospital (RR 0.40; 95% CI 0.29 to 0.54) (see online supplementary file 7). Applying a definition of catastrophic cost at 20% of annual household income, home-based management was associated with an 11.4% risk of catastrophic cost whereas hospital-based management was associated with a 62.0% risk of catastrophic cost (RR 0.16; 95% CI 0.08 to 0.41) (see online supplementary file 7). The reduction in risk of catastrophic cost was seen irrespective of wealth quartile, gender or HIV status.

10.1136/thoraxjnl-2018-212675.supp7 Supplementary data

## Discussion

This trial failed to meet target recruitment and was therefore insufficiently powered to demonstrate non-inferiority of the intervention; however, the available data do not demonstrate a difference in clinical outcomes between people receiving injectable treatments for TB delivered by lay carers and the traditional hospital-based model of care. Additionally, catastrophic patient costs were largely avoided by home-based care.

The RD between the two groups in the primary outcome was only 3%. However, the CIs around this estimate reached 9%; therefore, non-inferiority cannot be concluded at the predefined margin of 6%. These figures are open for discussion. First, a non-inferiority margin of 6% was conservative, with many other trials setting margins of 10%–15%.^21–24^ Second, the point estimate of 3% difference in risk is low and the absolute risk of patients failing to complete 2 months of home-based retreatment was only 7%, much lower than previous estimates of death or loss to follow-up during the first 2 months of TB retreatment.

There were seven deaths in the home-based arm of the study, compared with only two in the hospital arm. The mortality rate was not statistically different between the two groups; however, the circumstances of these deaths still need to be carefully examined. A higher mortality may have been as a direct consequence of guardians administering injections, but none of the deaths were related to unsafe administration of an intramuscular injection such as sepsis. Additionally, there is no evidence that adherence to treatment in the home-based arm was a problem, as both measures of adherence suggested very few missed doses of streptomycin; the rate of sputum culture conversion at 2 months was similar in both groups; and all of the deaths were due to conditions other than TB. Another possible reason for an increased death rate in the community is that patients at home had less medical attention during the intensive phase of treatment. Some evidence for this may be suggested by the higher number of adverse events in the hospital arm. However, any prescription of medication by a physician was classified as an adverse event and it is well recognised that overprescription of antibiotics and other drugs by clinicians to patients in hospital is common.^25 26^ An indirect effect of home-based care also seems unlikely given that, of the seven deaths in people randomised to the home-based arm, six happened only following readmission to hospital. In 5 out of 6 of these patients, the death took place only after they had been receiving appropriate treatment in hospital for at least 7 days. If this model of care is to be more widely adopted, it will require close ongoing evaluation.

There was no difference between the two study arms in terms of successful 8-month completion of TB treatment (80.7% in the home-based model vs 77.2% in the hospital-based model), which is operationally the most significant outcome from the perspective of global TB control. Additionally, the rate of 8-month cure was significantly higher (49.4% vs 32.9%, RR 1.50 (1.02 to 2.20; p value 0.039)) and the rate of 2-month culture conversion was non-significantly higher in the home-based arm (91.3% in the home vs 75.0% in the hospital). The mechanisms by which home-based care may be contributing to improving some outcome indicators may be through more ownership of health decisions, fewer economic consequences and ultimately better adherence to treatment.^27 28^

The study has a number of limitations. Most importantly, the trial was stopped early because of futility, which impacts the strength of the data. Efforts had been made to predict the expected event rate by conducting a review of the QECH TB register prior to starting the study, but the observed event rate was lower than expected. It is likely that the main reason for this was the requirement for participants to have a guardian able and willing to undertake delivery of the injectable. This resulted in exclusion before randomisation of 102 patients (see figure 1). Patients without guardians or with guardians unwilling to take on the responsibility of injecting may have been less likely to have stable lifestyles. In this way, the trial probably recruited a more stable cohort of patients than those assessed in the record review. Additionally, it is common for outcomes in a trial setting to be better than in routine practice due to increased input from the study team.^29 30^ Although a proportion of patients were not recruited because they were never clinically stable enough to be discharged, it was never intended for the intervention to be applicable to all patients prescribed TB retreatment, and even under routine conditions only those well enough to go home would be considered for home-based management. Finally, the study was conducted at only two sites in Malawi, both in large urban areas, and so results are not necessarily generalisable to other contexts.

The trial also has a number of strengths. As far as possible, the study was pragmatic and provides data for policy-makers that are relevant to routine operational conditions^31 32^, for example: reviews conducted by research staff with experience equivalent to that of a health surveillance assistant^27^; eligibility criteria including all patients clinically fit for discharge without additional investigations which would not be available in routine practice; no therapeutic drug monitoring to assess adherence in the community; and a primary outcome measure which is routinely recorded in TB reporting systems. Additionally, a trial-based economic evaluation provides robust data demonstrating that home-based management is associated with significant reductions in both provider and user costs, and qualitative evaluation (published elsewhere^33^) demonstrates the acceptability of lay carers delivering injectables.

The reduction in provider costs from US$1100 to US$489 potentially has an enormous impact on health systems in countries like Malawi, where the total expenditure on health per capita is US$93.^28^ Additionally, the intervention reduced the risk of catastrophic household costs ≥10% by 60%, and the risk of catastrophic household costs ≥20% by 84%. There are now strong data demonstrating that household catastrophic costs in relation to TB are associated with poorer clinical TB outcomes,^34^ and one of the targets of the WHO EndTB strategy is that ‘no TB-affected families should experience catastrophic costs due to TB’.^35^ This study was underpowered for clinical outcome, and another large randomised controlled trial would be costly and time consuming. However, the data demonstrate compelling health economic benefits for both providers and users. If TB programmes consider adopting this model, further data should be collected under operational conditions in order to monitor ongoing clinical safety and economic benefit.

Recent guidelines encourage a move away from the use of long-term-injectable agents for TB. However, the complete phase out of Category II regimen is still dependent on the availability of rapid molecular-based drug-susceptibility testing and resolution of the debate surrounding the best treatment for isoniazid monoresistant TB. Similarly, despite recent communications from WHO, the global use of fully oral regimens for all people with MDR-TB is still some way off given the financial and operational issues which still surround these regimens.

If targets to reduce catastrophic household expenditure associated with TB are to be met, the issue of how to deliver long-term injectable agents to stable patients in the community needs to be addressed. This study presents a novel method of administering injections to patients in their homes as part of their long-term TB care. Training patient-nominated lay people to deliver injectables may offer a sustainable opportunity for home-based management of patients with recurrent or drug-resistant TB in resource-limited settings but further operational data to support the findings of this study are needed.
