# Supplementary material for: Delivery of long-term-injectable agents for TB by lay carers: pragmatic randomised trial
Source: Thorax. 2019 Nov 1;75(1):64–71. doi: 10.1136/thoraxjnl-2018-212675 (PMC6929921; doi:10.1136/thoraxjnl-2018-212675)
Supplement: Supplementary data [file thoraxjnl-2018-212675supp001.pdf]

53573

Form RROC_F1 First Competency Test

TB - RROC study

Study ID F101 [ ]

Write barcode number here [| | | | |]

Interviewer check the box if this page has any corrections ☐

Assessment			
F102	DRAW	Draws up sterile water into syringe	Yes ☐ (1) No ☐ (2)
F103	RECON	Correctly reconstitutes antibiotic in vial	Yes ☐ (1) No ☐ (2)
F104	WDRAW	Withdraws correct amount of antibiotic into syringe	Yes ☐ (1) No ☐ (2)
F105	CHANG	Changes needle before performing injection	Yes ☐ (1) No ☐ (2)
F106	BUTT	Correctly locates safe injection site	Yes ☐ (1) No ☐ (2)
F107	CLEAN	Cleans the skin	Yes ☐ (1) No ☐ (2)
F108	ADVAN	Correctly advances needle into muscle perpendicular to skin	Yes ☐ (1) No ☐ (2)
F109	FLASH	Withdraws to check for flashback of blood	Yes ☐ (1) No ☐ (2)
F110	ADMIN	Administers contents of syringe	Yes ☐ (1) No ☐ (2)
F111	SAFE	Washes hands, wears gloves and disposes of all materials safely	Yes ☐ (1) No ☐ (2)

Competency			
F112	COMP	Is the guardian competent to sefely administer intramuscular streptomycin?<br>***(to be competent, guardian must score 10/10)***	Yes ☐ (1) No ☐ (2)

Knowledge of side effects			
F113	KNOW	Is the guardian competent to recall possible complications of streptomycin injections?	Infection at the place of injection ☐ (1)<br>Leg going weak ☐ (2)<br>Not passing urine ☐ (3)

Form completion			
Staff ID<br>F114 IID	[| |]	Date completed<br>F115 DCOM	[D D] [M O N] [2 0 1 Y]

Cohen DB, *et al. Thorax* 2019; 75:64–71. doi: 10.1136/thoraxjnl-2018-212675
