# Supplementary material for: Delivery of long-term-injectable agents for TB by lay carers: pragmatic randomised trial
Source: Thorax. 2019 Nov 1;75(1):64–71. doi: 10.1136/thoraxjnl-2018-212675 (PMC6929921; doi:10.1136/thoraxjnl-2018-212675)
Supplement: Supplementary data [file thoraxjnl-2018-212675supp002.pdf]

Supplementary file 2. Home monitoring form

Month	1	2	3	4	5	6	7	8	9	10	11	12	13	14	15	16	17	18	19	20	21	22	23	24	25	26	27	28	29	30	31

Ulendo	Tsiku	Date	Nthawi
1			
2			
3			
4			

Ngati pangakhale zovuta:

Lolemba kufikira Lachisanu 8 koloko mpaka 5 koloko, phone:

[redacted]

Nthawi ina ili yonse:
Pitani ku Ngozi kapena ku Wodi 3A ku chipatala cha
Queen Elizabeth central Hospital.
