# Supplementary material for: Delivery of long-term-injectable agents for TB by lay carers: pragmatic randomised trial
Source: Thorax. 2019 Nov 1;75(1):64–71. doi: 10.1136/thoraxjnl-2018-212675 (PMC6929921; doi:10.1136/thoraxjnl-2018-212675)
Supplement: Supplementary data [file thoraxjnl-2018-212675supp003.pdf]

Supplementary file 3. Adverse Event definitions

**Adverse event (AE)**	Any untoward medical occurrence in a study participant
Clinical event	Any illness or clinical injury, including possible infection at an injection site, possible sciatic nerve injury, possible renal impairment, possible hearing loss, prescription of any new medication
PEP event	Any possible blood-borne infection exposure occurring in relation to administration of streptomycin, including needle-stick injuries, which may or may not require post exposure prophylaxis (PEP) for HIV
Adherence event	Any situation in which the guardian does not administer daily streptomycin injections, and includes missed doses identified by self-report or vial count at routine review, or the guardian being unavailable to give the dose for any other reason (e.g. travelling away)
Default event	Any missed routine follow-up visit
**Serious Adverse Event (SAE)**	Any adverse event that:<br>i. Results in death<br>ii. Is life-threatening<br>iii. Requires hospitalisation or prolongation of existing hospitalisation (including readmission because of any clinical, adherence, safety or PEP event)<br>iv. Results in persistent or significant disability or incapacity (including sciatic nerve injury or ototoxicity)<br>v. Results in a patient being lost to follow up<br>vi. Results in any caregiver requiring Post Exposure Prophylaxis following possible exposure to blood-borne virus
**Unexpected Serious Adverse Event (USAE)**	Any SAE that, for any reason, is deemed to be unexpected and requires expedited review
