# Supplementary material for: Delivery of long-term-injectable agents for TB by lay carers: pragmatic randomised trial
Source: Thorax. 2019 Nov 1;75(1):64–71. doi: 10.1136/thoraxjnl-2018-212675 (PMC6929921; doi:10.1136/thoraxjnl-2018-212675)
Supplement: Supplementary data [file thoraxjnl-2018-212675supp004.pdf]

**STANDARD OPERATING PROCEDURE**
**ADVERSE EVENT MANAGEMENT AND REPORTING**

TB-RROC Study

## PURPOSE

- To ensure the safety and welfare of all participants in the TB-RROC Study.
- To minimise the risk of patients defaulting from treatment by actively following up those who miss scheduled appointments.
- To minimise the risk to patients and guardians from clinical events related to receiving TB retreatment in the community and in hospital.
- To ensure accurate recording of all Adverse Events.
- To ensure timely and efficient reporting of Severe Adverse Events.

## SCOPE

All Adverse Events and Serious Adverse Events occurring within the TB-RROC Study will be managed according to these standardised procedures.

## DEFINITIONS

**Adverse Event (AE)**
An Adverse Event is defined as any untoward medical occurrence in a study participant. For the purposes of this study, there will be four categories of adverse event:
<u>Clinical event</u>
This includes possible infection at an injection site, possible sciatic nerve injury, possible renal impairment, possible hearing loss, prescription of any new medication and death.
<u>PEP event</u>
This refers to any possible blood-borne infection exposure occurring in relation to administration of streptomycin, including needle-stick injuries.
<u>Adherence event</u>
This refers to any situation in which the treatment supporter does not administer daily streptomycin injections, and includes missed doses identified by self-report or vial count at routine review, or treatment supporters being unavailable to give the dose for any other reason (e.g. travelling away).
<u>DNA event</u>
This refers to any missed routine follow up visit.

**Serious Adverse Event (SAE)**
A Serious Adverse Event is defined as any Adverse Event that:
i. Results in death.
ii. Is life-threatening.
iii. Requires hospitalisation or prolongation of existing hospitalisation (including readmission because of any clinical, adherence, safety or PEP event).



Cohen DB, *et al. Thorax* 2019; 75:64–71. doi: 10.1136/thoraxjnl-2018-212675

iv. Results in persistent or significant disability or incapacity (including sciatic nerve injury or hearing loss).
v. Results in a patient being lost to follow up.
vi. Results in any caregiver requiring Post Exposure Prophylaxis following possible exposure to blood-borne virus.

**Unexpected Serious Adverse Event (USAE)**
An Unexpected Serious Adverse Event is defined as any SAE that, for any reason, is deemed to be unexpected and requires expedited review.

## Personnel Responsible

- All study team members: responsible for identification and reporting of Adverse Events.
- Study Nurse: responsible initial assessment and management of Adverse Events, reporting and liaising with the PI.
- PI: responsible for secondary assessment of all Adverse Events by discussion with Study Nurse; and assessment of all Serious Adverse Events by reviewing all patients who have an SAE, together with the Study Nurse.

## Procedures

The procedures for management and reporting of adverse events are summarised in figure 1.

### 1. Identification of adverse events

Three methods of presentation may identify adverse events:
- Routine follow up visit
- Participant initiated contact with the study team between scheduled routine reviews
- Participant does not attend scheduled routine review

### 2. Management and documentation of adverse events

Clinical event identified

- After identifying a clinical event, the study nurse will liaise with the PI.
- Appropriate action will be taken depending on the clinical picture.
- If a clinical event is identified, the case notes will be reviewed by a third independent clinician who will decide if the clinical event meets the definition of an SAE.
- If there is discrepancy between the SAE decision of the PI and the third clinician, the final classification will be resolved by consultation.

- If the study nurse identifies hearing loss compared to baseline, the PI will repeat the CALFRAST test. If the two tests are not in agreement, the third independent clinician will repeat the CALFRAST test.
- The following form will be completed:
  - RROC_S: Adverse event form (Section 1, section 2 and section 6 if appropriate)
- If the clinical event results in readmission to hospital, death, life-threatening illness, permanent or significant disability or incapacity, this will constitute an SAE.

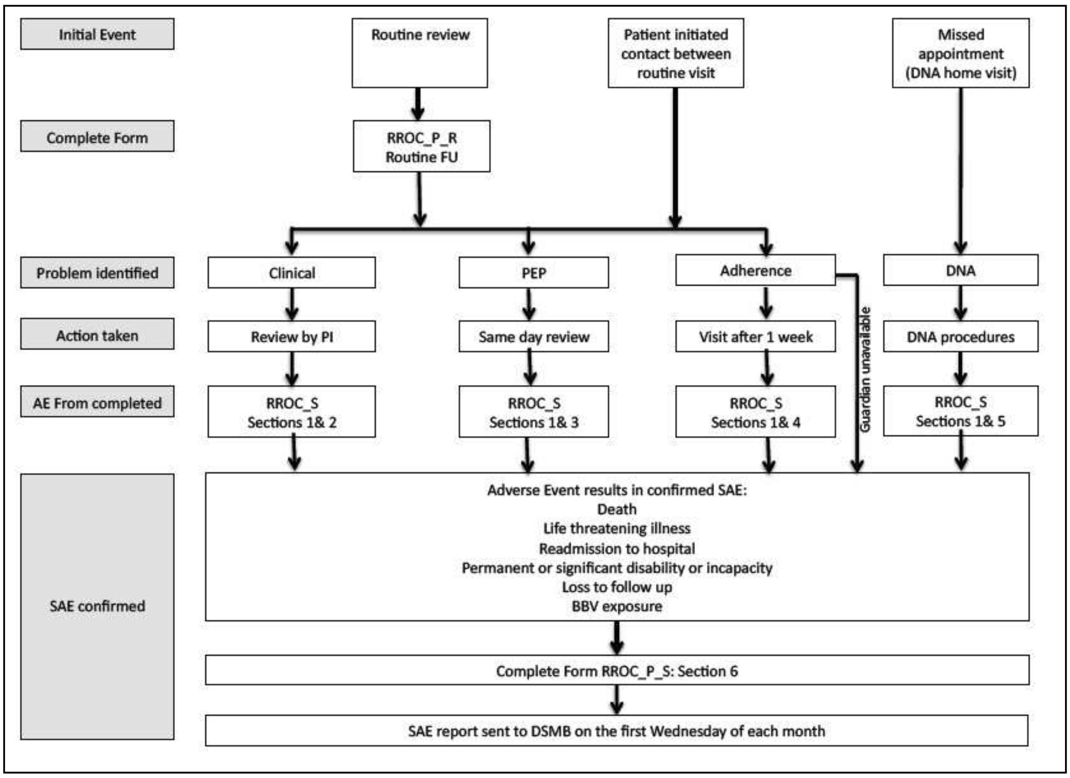


**Figure 1. Adverse Event procedures**

<u>PEP event identified</u>

- The management of exposures to HIV will follow the MoH recommendations.
- Without any intervention, the risk of transmission of HIV following a needle-stick injury is low (0.3%; or 3 in 1000 exposures).
- 'Post Exposure Prophylaxis' (PEP) refers to the treatment of exposures to HIV using antiretroviral drugs (ARVs). PEP is not a therapy of a disease but a preventive treatment.
- ARVs started immediately after exposure to HIV may prevent HIV infection, although this protection is not 100%.
- ARVs should be started as soon as possible. It is reasonable to start PEP up to 72 hours after the exposure, but the sooner it is started, the more effective it is.
- PEP is safe in pregnancy and breastfeeding.



Cohen DB, *et al. Thorax* 2019; 75:64–71. doi: 10.1136/thoraxjnl-2018-212675

- Risk of transmission after exposure to HIV can be classified according to risk (see table one)
- Nurses who have a needle-stick injury whilst administering streptomycin will be encouraged to report this to the study team. They will follow local PEP procedures.
- All participants will be instructed to telephone the study team following a needle-stick injury which occurs during the administration of streptomycin at home.
- If a participant telephones reporting a needle-stick injury, the study team will telephone them back.
- The following instructions will be given:
    - Use soap and water to rinse any wound or skin site in contact with the blood.
    - Do not use bleach, antiseptics or other caustic substances.
    - The patient and exposed person should come to the hospital as soon as possible that day.
- Once the participants arrive at the hospital, the following will take place:
    - If the HIV status of the patient is not known, HIV testing should be carried out after normal counselling procedures, using rapid HIV tests or coded lab forms. If the HIV status of the source person cannot be determined immediately, PEP should be started as soon as possible.
    - If the HIV status of the exposed person is unknown, they should undergo HIV testing as soon as possible, following the normal counselling procedures. If the exposed person is HIV positive, PEP is contraindicated; they should be referred for further care at their local ART clinic.
    - If the exposure is assessed to be high risk, a prescription for 30 days of ARV tablets will be provided to the exposed person as soon as possible. There will be a supply of PEP medications in the research or nurses office on the TB ward.
    - The following ARV schedules will be used (see figure 2):
        - **Standard PEP:**
          Zidovudine/Lamivudine (AZT/3TC)
        - **Alternative PEP if exposed person is anaemic (Hb <8.0mg/dl):**
          Stavudine/Lamivudine (d4T/3TC)
        - **Alternative PEP if patient is on first line or alternative first line ART regimen:**
          Zidovudine/Lamivudine (AZT/3TC) *or*
          Stavudine/Lamivudine (d4T/3TC) *plus* Aluvia
- The following forms will be completed:
    - PEP form
    - RROC_S: Adverse event form (Section 1, section 2 and section 6 if appropriate)
- The study doctor will review all HIV exposure events as soon as possible after the event.
- The study doctor will review the exposed person 2 weeks following exposure, and their Hb will be checked.
- The study nurse will review the exposed person 4 months following exposure, and the exposed person will undergo repeat HIV testing.



Cohen DB, *et al. Thorax* 2019; 75:64–71. doi: 10.1136/thoraxjnl-2018-212675

| | Substance | Type of contact | Source person |
|---|---|---|---|
| Risk | • Blood<br>• Semen<br>• Vaginal fluid<br>• Cerebro-spinal fluid<br>• Pleural fluid<br>• Amniotic fluid<br>• Synovial fluid<br>• Ascites fluid | • Skin penetrated with contaminated needle (hollow or non-hollow)<br>• Large amount of substance on mucous membrane<br>• Sexual intercourse no condom<br>• Risk substance on lacerated skin / open wound | • Known HIV infected<br>• Unknown HIV status<br>• Recently tested negative (may be in window period) |
| No Risk | • Urine<br>• Stool<br>• Pus<br>• Tears<br>• Saliva<br>• Sputum<br>• Nasal secretions | • Risk substance on intact skin | |

**Table1: Classification of risk of transmission after exposure to HIV**

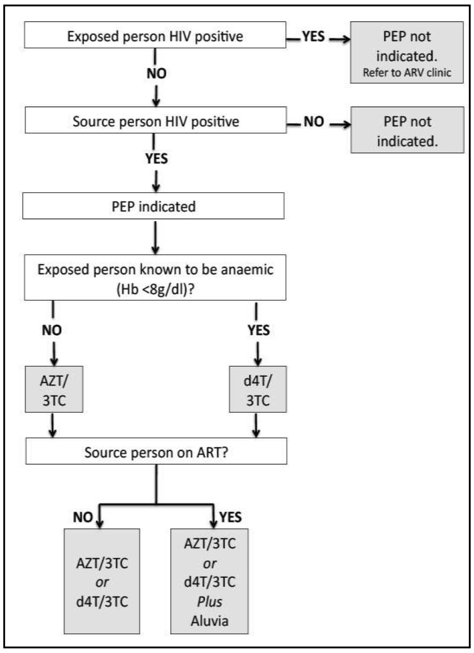


**Figure 2. PEP flowchart**

Adherence event identified

**All patients**

*Missed doses*

- If, at routine review, there is a discrepancy between the number of used vials and the number of days since the last visit, or the participant reports missed doses, the nurse will do adherence counselling with the participants.

**For patients managed in the community**

- If the patient has missed more than 2 doses since the last visit, the participants will be visited after one week and the following form will be completed:
  - RROC_S: Adverse event (Section1, section 4, section 6 if SAE identified)
- At the one-week follow up visit, if the patient has missed more than 1 dose since the last visit, the patient will be readmitted to the TB ward, and this will therefore constitute an SAE.

*Treatment supporter unavailable*

- If a treatment supporter is no longer available to administer daily streptomycin injections, this automatically results in an adherence event and will require readmission to the TB ward, and this therefore constitutes an SAE.

Participants DNA scheduled routine review

- If both participants are not available at the scheduled routine follow-up visit, any information from household members or neighbours will be used to try and determine the reason for the missed visit, and rearrange another visit.
- If the visit cannot be rescheduled in this way, the following people will be telephoned in order to try and find the patient and rearrange a meeting with the patient:
  - The patient
  - The treatment supporter
  - The contacts provided
- The following form will be completed:
  - RROC_S: Adverse event (Section 1, section 5 and section 6 if appropriate)
- If a successful follow up visit is not completed within 2 weeks of the missed appointment, this will constitute a loss to follow up event, which is automatically classified as an SAE.

SAE confirmed

- The PI, in conjunction with the study nurse, will review the case records of each participant identified as having an SAE.
- The following form will be completed:
  - RROC_S: Adverse event form (Section 6)

**3. Reporting of adverse events**

- Monthly reports will be compiled by the study statistician (who will be blinded to participant study group) and sent to the DSMB for review.
- Reports will include
  - The total number of adverse event forms (Clinical/PEP/Adherence/DNA) in each group.
  - A brief description of each SAE.



Cohen DB, *et al. Thorax* 2019; 75:64–71. doi: 10.1136/thoraxjnl-2018-212675
