# Supplementary material for: Delivery of long-term-injectable agents for TB by lay carers: pragmatic randomised trial
Source: Thorax. 2019 Nov 1;75(1):64–71. doi: 10.1136/thoraxjnl-2018-212675 (PMC6929921; doi:10.1136/thoraxjnl-2018-212675)
Supplement: Supplementary data [file thoraxjnl-2018-212675supp005.pdf]

Supplementary file 5

Methods used in the economic evaluation of the TB-RROC trial

For each participant, we estimated the total health provider cost by multiplying the healthcare resources used by the unit cost for the resource. Structured questionnaires were used to prospectively record healthcare resources during a defined period. Unit costs for healthcare resources were either estimated through primary costing studies or extracted from primary hospital costing study undertaken around the same time (1). The health provider cost for the home-based intervention included: 1) costs of training staff; 2) costs of training guardians to deliver intramuscular streptomycin; 3) costs of routine follow up; 4) costs of unscheduled attendances (adverse events); and 5) costs of TB drugs.

An interviewer-administered questionnaire was developed based on previous approaches to estimate the direct and indirect costs incurred by participants (2). Healthcare in Malawi is provided free; however patients sometimes purchase additional drugs or investigations from the private sector: these costs were termed direct medical costs. Direct non-medical costs included the cost of transportation, food and drinks and other costs incurred either during hospital admissions or when visiting a healthcare facility to receive care. Indirect costs (lost income) were estimated by multiplying time off work by self-reported income (3).

The analysis performed was a cost-consequence analysis and is presented as the incremental cost-difference per patient completing the intensive phase of retreatment.

We also estimated the proportion of participants in each arm who incurred catastrophic healthcare expenditure. Catastrophic healthcare cost was primarily defined as total user costs (sum of direct medical, direct non-medical and indirect costs) amounting to ≥10% of annual household income (4-6). A limited sensitivity analysis was performed using a threshold of ≥20% annual household income. Costs were recorded as the expenses incurred by the trial in Malawi Kwacha, Pounds Sterling or US dollars, and the year the cost was incurred was noted. The costs were adjusted to the year of reporting using the Gross Domestic Product deflator index, provided by the World Bank. All costs were converted into 2014 US Dollars using the market exchange rate. To examine differences in costs between the trial arms we used non-parametric bootstrap methods to derive 95% confidence intervals for mean cost differences.

1. Maheswaran H, Petrou S, Cohen D, MacPherson P, Kumwenda F, Lalloo DG, et al. Economic costs and health-related quality of life outcomes of hospitalised patients with high HIV prevalence: A prospective hospital cohort study in Malawi. PLoS One. 2018;13(3):e0192991.
2. Tool to estimate patient costs. StopTB partnership; 2008.
3. Pritchard C, Sculpher M. Productivity costs: principles and practice in economic evaluation London: Office of Health Economics; 2000.
4. Ukwaja KN, Alobu I, Abimbola S, Hopewell PC. Household catastrophic payments for tuberculosis care in Nigeria: incidence, determinants, and policy implications for universal health coverage. Infect Dis Poverty. 2013;2(1):21.
5. Barter DM, Agboola SO, Murray MB, Barnighausen T. Tuberculosis and poverty: the contribution of patient costs in sub-Saharan Africa--a systematic review. BMC Public Health. 2012;12:980.
6. Ranson MK. Reduction of catastrophic health care expenditures by a community-based health insurance scheme in Gujarat, India: current experiences and challenges. Bull World Health Organ. 2002;80(8):613-21.
