# Supplementary material for: Delivery of long-term-injectable agents for TB by lay carers: pragmatic randomised trial
Source: Thorax. 2019 Nov 1;75(1):64–71. doi: 10.1136/thoraxjnl-2018-212675 (PMC6929921; doi:10.1136/thoraxjnl-2018-212675)
Supplement: Supplementary data [file thoraxjnl-2018-212675supp006.pdf]

Supplementary file 6. Provider costs

a) Home-based management

	Mean costs (95% CI) 2014 US dollars n=32
**Training guardians**	
Days of admission	301.0 (233.0-369.1)
Nurse time	7.5 (5.8-9.3)
Gloves	1.2 (0.8-1.5)
Syringes	0.4 (0.3-0.5)
Needles	0.8 (0.7-1.0)
Water for injection	0.7 (0.6-0.9)
Alcohol swabs	0.2 (0.2-0.3)
Training materials	1.2
Overhead costs	1.3 (1.0-1.6)
TOTAL TRAINING COSTS	314.3 (245-383.0)
**Routine follow up**	
HSA time	9.7 (7.8-11.6)
HSA transport	7.4 (5.4-9.5)
Gloves	5.6 (5.0-6.2)
Syringes	2.7 (2.4-3.0)
Needles	3.1 (2.8-3.4)
Water for injection	4.6 (4.2-5.1)
Alcohol swabs	1.2 (1.0-1.3)
Sharps boxes	4.6 (4.3-5.0)
Overhead costs	4.9 (3.9-5.9)
TOTAL FOLLOW UP COSTS	43.9 (38.0-49.7)
**Adverse events**	
HSA time	0.1 (0.0-0.2)
Nurse time	0.01 (0.0-0.1)
Junior clinician time	0.1 (-0.1-0.4)
Senior doctor time	0.5 (0.1-1.0)
Drugs	3.7 (-2.2-10.0)
Investigations & procedures	6.8 (-1.5-15.1)
Ward stay for readmission	22.2 (0.0-56.1)
Airtime	0.3 (0.0-0.6)
Overhead costs	0.1 (0.0-0.2)
TOTAL ADVERSE EVENT COSTS	34.0 (0.0-75.7)
**TB drugs**	85.4 (80.9-89.8)
**ART**	20.5 (19.4-21.6)
**TOTAL PROVIDER COSTS**	**498.0 (425.6-570.4)**

b) Hospital-based management

	Mean costs (95% CI) 2014 US dollars n=33
Investigations & procedures	109.2 (68.5-149.9)
Drugs	114.0 (110.1-117-9)
Ward stay	895.6 (859.1-932.1)
**TOTAL PROVIDER COSTS**	**1100.3 (1040.8-1159.8)**
