# Supplementary material for: Delivery of long-term-injectable agents for TB by lay carers: pragmatic randomised trial
Source: Thorax. 2019 Nov 1;75(1):64–71. doi: 10.1136/thoraxjnl-2018-212675 (PMC6929921; doi:10.1136/thoraxjnl-2018-212675)
Supplement: Supplementary data [file thoraxjnl-2018-212675supp007.pdf]

Additional file 7a. Risk of incurring costs of ≥10% of household income according to study arm

	Total population	Hospital-based management	Home-based management	Risk Ratio
All participants	109/180 (60.6)	79/92 (85.9)	30/88 (34.1)	0.40 (0.29-0.54)
Wealth quartile				
First	21/41 (51.2)	17/19 (89.5)	4/22 (18.2)	0.20 (0.08-0.50)
Second	31/45 (68.9)	22/26 (84.6)	9/20 (45.0)	0.53 (0.32-0.89)
Third	28/46 (60.9))	20/25 (80.0)	8/21 (38.1)	0.48 (0.27-0.85)
Fourth	27/44 (64.3)	20/23 (87.0)	7/21 (33.3)	0.38 (0.21-0.72)
Gender				
Male	72/127 (56.7)	50/60 (83.3)	22/67 (32.8)	0.39 (0.27-0.57)
Female	37/53 (69.8)	29/32 (90.6)	8/21 (38.1)	0.42 (0.24-0.73)
HIV status				
Negative	13/35 (37.1)	9/12 (75.0)	4/23 (17.4)	0.23 (0.09-0.60)
Positive	96/145 (66.2)	70/80 (87.5)	26/65 (40.0)	0.46 (0.34-0.62)

Additional file 7b. Risk of incurring costs of ≥20% household income according to study arm

	Total study population	Hospital-based management	Home-based management	Risk Ratio
All participants	67/180 (37.2)	58/92 (62.0)	9/88 (11.4)	0.16 (0.08-0.41)
Wealth quartile				
First	13/41 (31.2)	13/19 (68.4)	0/22 (0.0)	NA
Second	17/45 (37.8)	14/26 (53.9)	2/20 (10.0)	0.17 (0.04-0.64)
Third	22/46 (47.8)	16/25 (64.4)	6/21 (28.6)	0.45 (0.20-0.93)
Fourth	15/44 (34.1)	14/23 (60.1)	1/21 (4.8)	0.07 (0.01-0.54)
Gender				
Male	42/127 (33.1)	36/60 (60.0)	6/67 (9.0)	0.15 (0.07-0.33)
Female	25/53 (47.2)	22/32 (68.8)	3/21 (14.3)	0.21 (0.07-0.61)
HIV status				
Negative	7/35 (20.0)	7/12 (58.3)	0/23 (0.0)	NA
Positive	60/145 (41.4)	51/80 (63.8)	9/65 (13.9)	0.22 (0.12-0.41)
